# Unaffected Arm Muscle Hypercatabolism in Dysphagic Subacute Stroke Patients: The Effects of Essential Amino Acid Supplementation

**DOI:** 10.1155/2014/964365

**Published:** 2014-11-09

**Authors:** Roberto Aquilani, Mirella Boselli, Giuseppe D'Antona, Paola Baiardi, Federica Boschi, Simona Viglio, Paolo Iadarola, Evasio Pasini, Annalisa Barbieri, Maurizia Dossena, Andria Innocenza Bongiorno, Manuela Verri

**Affiliations:** ^1^Servizio di Fisiopatologia Metabolico-Nutrizionale e Nutrizione Clinica, Centro Medico di Montescano, IRCCS, Via per Montescano 31, 27040 Montescano, Italy; ^2^Unità di Riabilitazione Neuromotoria, Unità Gravi Cerebrolesioni Acquisite, Centro Medico di Montescano, IRCCS, Via per Montescano 31, 27040 Montescano, Italy; ^3^Laboratorio Universitario per lo Studio delle Attività Motorie nelle Malattie Rare (LUSAMMR), Via Ugo Foscolo 13, 27058 Voghera, Italy; ^4^Dipartimento di Medicina Molecolare, Università degli Studi di Pavia, Viale Taramelli 3/B, 27100 Pavia, Italy; ^5^Direzione Scientifica Centrale, Fondazione S. Maugeri, IRCCS, Via Salvatore Maugeri 10, 27100 Pavia, Italy; ^6^Dipartimento di Scienze del Farmaco, Università degli Studi di Pavia, Viale Taramelli 14, 27100 Pavia, Italy; ^7^Dipartimento di Biologia e Biotecnologie “Lazzaro Spallanzani,” Università degli Studi di Pavia, Via Ferrata 9, 27100 Pavia, Italy; ^8^Istituto Scientifico di Lumezzane (Brescia) Fondazione S. Maugeri, IRCCS, Via Mazzini 9 Lumezzane, 25065 Brescia, Italy

## Abstract

Alterations in muscle protein turnover of the unaffected side of stroke patients could contribute to physical disability. We investigated whether hypercatabolic activity occurred in unaffected arm muscle and whether supplemented essential amino acids (EAAs) could limit muscle hypercatabolism (MH). Thirty-eight dysphagic subacute stroke subjects (<3 months after acute event) (29 males + 9 females; 69.7 ± 11.4 yrs) were enrolled and randomized to receive 8 g/day EAAs (*n* = 19; EAA group) or isocaloric placebo (maltodextrin; *n* = 19, Plac group). Before randomization, all patients had their arterial (A) and venous (V) amino acids measured and muscle (A − V) differences calculated in the unaffected arm. Eight matched and healthy subjects served as controls. When compared to healthy controls, the entire stroke population showed significant muscle release (= negative value A − V) of the amino acid phenylalanine (phenyl-) indicating a prevalence of MH. Moreover, randomized EAA and Plac groups had similar rates of MH. After 38 days from the start of the protocol, the EAA group but not the Plac group had MH converted to balanced protein turnover or anabolic activity. We concluded that muscle protein metabolism of the unaffected arm of dysphagic subacute stroke individuals could be characterized by MH which can be corrected by supplemented EAAs.

## 1. Introduction

Strokes are the world's leading cause of disability. About one-third of stroke survivors are permanently disabled one year after the acute event [1, 2]. About two-thirds of patients do not completely recover after strokes [3], while one-third cannot walk without assistance [4]. Furthermore, in hemiparetic subjects who can still walk, gait efficiency is reduced and the energy cost of the gait is increased compared with efficient symmetric gait [5, 6].

In addition to the loss of central trophic effects [7, 8] and transsynaptic degeneration of lower motor neurons [9–11], poststroke skeletal muscle changes can also potentially contribute to disability. These changes include fibre-type shift in the paretic (= controlateral) side [12, 13], increased intramuscular fat (myosteatosis) substituting muscle tissue [14], spasticity [15], disuse [16, 17], malnutrition [18], and muscle unloading [19].

Subjects with subacute stroke (<3 months after acute event) [20, 21] were studied to find whether stroke disability could be due to altered protein turnover in unaffected muscles. Our hypothesis is based on a previous study which showed that skeletal muscles of subacute stroke patients could be hypercatabolic (i.e., protein degradation is higher than protein synthesis) due to a persistent systemic inflammatory state [22]. Muscle hypercatabolism (MH) of the unaffected side could contribute to patient disability by inducing a loss of both muscle mass and strength, that is, a state of sarcopenia [23]. Highlighting MH as early as possible could be clinically important in order to limit patient disability.

We also formulated another hypothesis in that MH could be attenuated or even converted to muscle anabolism by providing patients with essential amino acids supplements (EAAs) as these nutrients are potent promoters of protein synthesis. This fact has been amply documented in animal [24–27] and human studies, both in healthy subjects [28, 29] and in those suffering from chronic diseases associated with muscle depletion and different from stroke [30–33].

This study was therefore carried out on subacute stroke patients with the aim of highlighting protein turnover (i.e., anabolic and catabolic rate) of the unaffected arm to document whether EAA supplements could reduce MH.

## 2. Subjects and Methods

### 2.1. Population

Sixty-seven dysphagic subacute stroke patients (<3 months after acute cerebrovascular event) [20, 21] admitted to our rehabilitation centre were eligible for the study. Following our criteria, 11 were excluded for associated chronic heart failure, 1 for acute coronary syndrome, 4 for acute or chronic renal failure (creatinine clearance < 30 mg/100 mL), 1 for cancer surgery, 2 for pressure ulcer, 7 for diabetes (on oral hypoglycemic or insulin treatment), 2 for dysthyroidism, and finally 1 for being on steroid therapy. The reason for excluding these diseases was strictly related to their strong impact on muscle protein metabolism.

The remaining thirty-eight patients (29 males + 9 females; 69.7 ± 11.4 yrs) were enrolled in this randomized, double blind, placebo-controlled study. The reason for patient admission was due to rehabilitation for dysphagia and hemiplegia. All patients were bedridden and had been admitted from neurosurgery (34.2%), neurological or stroke units (52.6%), or other rehabilitation settings (13.2%). Cerebrovascular accident documented by computerized tomography was characterized by ischemia in 57.9% of patients or haemorrhagic injury in the remaining 42.1%. We pooled ischemic and haemorrhagic individuals because, in the rehabilitative phase of stroke, these two groups have similar metabolic, nutritional, functional profiles [22].

On the basis of computerized tomography or magnetic resonance imaging, the damaged stroke areas were classified in relation to the location of the ischaemic destruction as PACI (partial anterior circulation infarction; 23.7%), TACI (total anterior circulation infarction; 50%), or POCI (posterior anterior circulation infarction; 26.3%). These data are contained in Table 1 which also shows stroke severity and assessed mechanisms underlying swallowing abnormalities.

At admission, all patients were fed via percutaneous endoscopy gastrostomy (PEG; *n* = 30) or by oral modified diet (*n* = 8).

### 2.2. Procedures

Within two days of admission, after overnight fasting at 8 am, blood samples were taken from each patient to determine the following.

#### 2.2.1. Plasma Amino Acids

These substrates were determined both in arterial (radial artery) and in venous blood of the unaffected arm. Concentrations of free amino acids in the plasma were measured using an AminoQuant II amino acid analyser, based on the HP 1090 HPLC system, with fully automated precolumn derivatization, using both ortho-phthalaldehyde and 9-fluorenyl-methyl-chloroformate reaction chemistries according to the manufacturer's protocol. The results were obtained by injecting 1 *μ*L of the derivatized mixture and measuring absorbance simultaneously at 338 and 262 nm. Plasma concentrations were expressed as *μ*mol/L. Amino acid measurements were carried out as a comparison in eight healthy subjects matched for age (71 ± 4.5 years), sex distribution (6 M/2 F), and body mass index (22.3 ± 3.5 kg/m^2^).

*Calculations*

- Muscle protein metabolism. As described elsewhere [34], muscle protein overdegradation was estimated by the muscle release of the essential amino acid phenylalanine (phenyl-), whereas muscle protein synthesis was determined by muscle phenyl- uptake. Given that phenyl- is neither synthesized nor degraded in muscle tissue, any changes in the muscle uptake/release would reflect the total protein balance [35]. A negative phenyl- A − V (= release) signified an imbalanced protein metabolism with an excess of protein degradation over protein synthesis, whereas a positive phenyl- A − V (= uptake) indicated a predominance of protein synthesis. A phenyl- A − V of zero (no uptake/no release) indicated a balanced muscle protein metabolism.
- A − V differences of the other amino acids, total amino acids (TAAs), total essential amino acids (EAAs: valine, isoleucine, leucine, threonine, phenyl-, tryptophan, methionine, lysine), and branched-chain amino acids (BCAAs: valine, isoleucine, leucine) were calculated.

#### 2.2.2. Biomarkers of Body Inflammatory Status

- Serum levels of interleukin-6 (IL-6; normal value < 7 pg/mL) were determined in duplicate, using a high-sensitivity commercial sandwich enzyme-linked immunosorbent assay (ELISA) kit from Mabtech (Agilent Technologies GmbH, Boblingen, Germany);
- C-reactive protein (CRP; normal value < 0.3 mg/dL) was determined with an immune-turbidimetric method;
- acute-phase reactant proteins (haptoglobin, normal values 30–200 mg/dL; *α*-1 globulin system, normal value 0.21–0.35 g/dL) and non-reactant proteins (albumin, normal values 4.02–4.76 g/dL; prealbumin, normal values 18–30 mg/dL and transferrin, normal values 202–364 mg/dL) were evaluated.

#### 2.2.3. Plasma Lactate Concentrations

These were measured with enzymatic tests following procedures recommended by the manufacturer (Siemens Diagnostic, Germany). The normal value is 0.6–2.2 mmol/L.

#### 2.2.4. As Part of Routine Assessment, Patients Had the Following Variables Measured

- Anthropometric characteristics: body weight (BW, kg), found using a mechanical weight lifter, and height (m), calculated from knee height [36]. Body mass index (BMI) was calculated as kg/m^2^. Patients (or their caregivers) were asked for their preacute BW. Loss of actual BW in relation to habitual (preacute) BW > 5%, that is, actual/habitual BW < 95%, was considered an index of significant undernutrition;
- biohumoral measurements: routine variables, including serum protein electrophoresis.

#### 2.2.5. Functional Status

This was evaluated using functional independence measure (FIM) [37]. This test is routinely used by the centre's neurorehabilitative physician. The FIM is an 18-item scale that measures patient independence in feeding, grooming, dressing, toileting, mobility, and cognition. A score of 126 indicates complete functional independence.

#### 2.2.6. Dysphagia

Identification of dysphagia was carried out clinically for the entire population. In case of positive or uncertain diagnosis, the patients underwent a video fluoroscopy examination. The severity of the dysphagia was evaluated using the Dysphagia Outcome and Severity Scale (DOSS), a 7-point scale developed to systematically rate the functional severity of dysphagia [38]. The score range was 1–7, where level 1 denotes severe dysphagia, level 2 moderately-severe dysphagia, level 3 moderate-dysphagia, level 4 mild-to-moderate dysphagia, level 5 mild-dysphagia, level 6 within functional limit/modified independence, and level 7 normal under all situations.

#### 2.2.7. Nutritional Intake

For self-feeding patients (*n* = 8) on a modified diet, a 3-day alimentary diary was kept by the rehabilitation nurses, who had been previously trained* ad hoc*. The nurses recorded the type and weight of cooked or uncooked food selected by patients from the hospital's catering menu on a diet sheet for 3 days both before and after the patients' meals. The amount of food actually ingested was converted (by Roberto Aquilani of the paper) to its raw equivalent when necessary, using appropriate tables [39]. Nutritional analysis, carried out using a computer program designed by this group [40], was used to calculate actual ingested calories and macro-/micronutrients. The nutritional intake from pharmaceutical formula of the patients with PEG (*n* = 30) was calculated from nutritional composition reported in the formula label.

#### 2.2.8. Rehabilitation Therapy

All patients received rehabilitative treatment adapted to each individual patient. Briefly, rehabilitation consisted of therapeutic exercise with a personal physiotherapist for 60 minutes, five days a week. The exercise included passive, active, and active-assistive range-of-motion exercise coordination, facilitation techniques of the controlateral limbs, trunk exercise, active exercises of the unaffected limbs, and ambulation with assistive devices or support. The number or repetition in exercise and walking distance was increased as the physical performance of the patients progressed. Speech therapy, occupational therapy (activities of daily living, vocational, perceptual, and functional activity training), and recreational activity were also performed depending on individual needs.

For dysphagia rehabilitation, attempts were made to provide patients with a DOSS levels ≥ 3 with a modified diet as well as to teach safe swallowing postural changes. For the diet, pureed, homogeneous, and cohesive foods were initially used with a gradual progression to food with nearly normal texture for individuals whose swallowing dysfunction progressively improved.

Postural changes during meals usually consisted of patients adapting techniques, which reduced the risk of aspiration. These included, for example, head rotation to the affected side, tilting of the head to the stronger side, chin tuck, and chin up movements.

For patients with DOSS < 3, attempts were made for oral transition after video fluoroscopic and/or after speech pathologists' assessments. If patients could safely eat at least two-third of their prescribed calories (1500 kcal/d), then tube feeding was discontinued.

### 2.3. Patient Randomization

**Figure 1:**
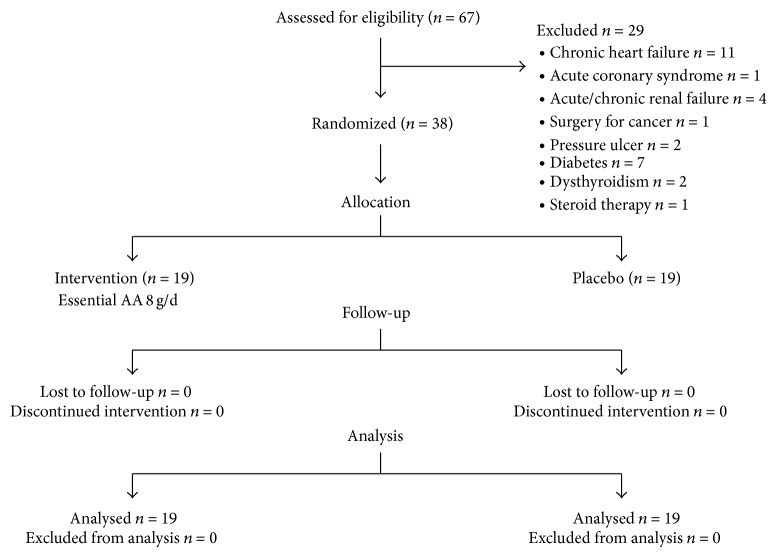
Flow diagram of a trial supplementation with EAAs mixture versus placebo to treat dysphagic stroke patients. The diagram includes the number of patients analyzed for the main outcome (unaffected arm muscle hypercatabolism).

After completing all these procedures, patients were assigned to treatment according to a randomized allocation procedure (Figure 1). A randomization list was generated using SAS statistical software (SAS Institute, Cary, NC). A and B were the identifiers of the blinded treatment. The list was made available both to the physician (Mirella Boselli) and to hospital pharmacists. The physician sequentially allocated patients to treatment A or B according to a randomization list. The first author (Roberto Aquilani), who interpreted all results, was blinded to the patients' allocation. The experimental group (EAA group) received nutrition mixture supplement that provided 8 g of EAAs/d (Aminotrofic, Professional Dietetics, Milan, Italy; Table 2; 4 g in the morning + 4 g in the afternoon diluted in half a glass of water until patient discharge). The placebo group (Plac) was given a similar isocaloric product containing maltodextrin instead of EAAs. Rehabilitation nurses assisted each patient with their oral diet during placebo or EAA intake to be sure of the patients' compliance.

The nurses were blinded to the type of supplementation (maltodextrin or EAAs); the packets containing the products were identical but numbered as 1 or 2. The contents were known only to the physician (Mirella Boselli) and pharmacists (1 = placebo; 2 = EAAs). The product content in packets 1 and 2 had a similar colour and taste. For patients receiving enteral nutrition (EN), the aqueous solution of EAAs was supplied through the feeding tube (percutaneous endoscopy gastrostomy). The study lasted 38 ± 4 days from the randomization procedure.

Amino acids, inflammation markers, and anthropometric and functional status measures were all repeated at the patients' discharge from rehabilitation (42 ± 4 days from admission). The study was approved by the Ethical-Technical Scientific Committee of the Institute. Written informed consent was obtained from participants or, whenever applicable, from their care-givers, after the nature of the study had been fully explained.

### 2.4. Statistical Analysis

Descriptive statistics were carried out for all recorded variables, reporting means, and standard deviations for quantitative variables and distribution frequencies for qualitative variables. Chi-squared test was used for categorical variables. Repeated measurement analysis of variance was used to assess any trend differences over time between patients on EAAs or Plac. Baseline differences between groups (EAAs and Plac) and differences in amino acid profiles between the entire stroke population at the admission to rehabilitation and healthy controls were tested by means of unpaired student* t*-test. Statistical significance was set at *P* < 0.05.

## 3. Results

All patients who entered this study were randomized to receive essential amino acid treatment (EAAs) or placebo (Plac; maltodextrin) (Figure 1).

### 3.1. Unaffected Arm Muscle Protein Turnover

Table 3 shows arterial amino acid concentrations and muscle amino acid arteriovenous differences (A − V) encountered for both stroke patients at admission to rehabilitation and healthy subjects.

The results showed that muscle protein metabolism of the unaffected side was prevalently in a hypercatabolic state (MH) due to excess of protein catabolism over protein synthesis indicated by muscle release of phenyl-. This was significantly different (*P* < 0.03) from healthy subjects whose muscle protein metabolism was in equilibrium. In addition to phenyl-, patients released significant amounts of asparagine, threonine, leucine, alanine, and taurine.

Regarding arterial amino acid concentrations, stroke patients had higher levels of serine, methionine, phenyl-, isoleucine, leucine, and lysine but lower concentrations of aspartic acid, asparagine, glutamine, alanine, taurine, and tryptophan. A subanalysis of patients, divided into type of cerebrovascular accident (ischaemic or haemorrhagic), revealed similar results.

Table 4 shows the amino acid profiles of the two patient subgroups randomized to receive EAAs or Plac, both at admission to and discharge from rehabilitation. At admission, the two subgroups had no significant difference in the MH rate (= phenyl-release) and in the other amino acid and total amino acid (TAA) A − V differences. Arterial concentrations of individual amino acids of TAAs and of EAAs were similar for both EAA and Plac.

At discharge, EAA but not Plac patients normalized their protein metabolism in the unaffected arm. Indeed, the release of phenyl- shifted to muscle uptake in treated patients but remained virtually unchanged in Plac patients. This difference in the time course of (A − V) phenyl- was significant (interaction, *P* = 0.02).

**Figure 2:**
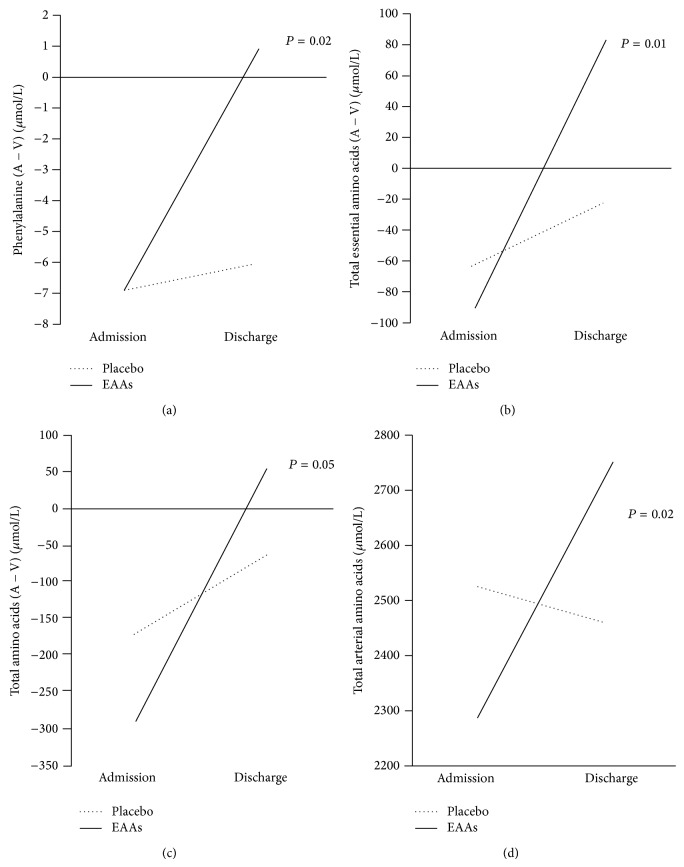
Time courses of phenyl-, total essential amino acid, total amino acid (A − V) differences, and total arterial amino acid levels of stroke population. The point 0 indicates no uptake/no release. For clarity, standard deviations are omitted (see Table 4).

We can see from Figure 2 that the discrepancy between the two subgroups also entailed arterial TAAs (*P* = 0.02), TAAs (A − V) (*P* = 0.05), EAAs (A − V) (*P* = 0.01), and BCAAs (A − V) (*P* = 0.05, not shown in the figure). Indeed, of all measured amino acids, 49% were taken up by EAA subjects, while only 23.2% by Plac ones (*P* < 0.001). The time courses of A − V differences between the two groups were also different for aspartic acid, histidine, asparagine, glycine, taurine, and tyrosine, released more in Plac than in the EAA groups. In the latter patients, aspartic acid was not released/not taken up.

### 3.2. Other Study Variables 

Table 5 shows demographic, anthropometric, neurofunctional, and biohumoral characteristics as well as nutritional intakes of stroke patients both as an entire group and as two subgroups after randomization both at admission and discharge. At admission, all subjects were malnourished due to postevent weight loss compared to their habitual BW (−9.7%). The patients were inflamed as shown by high serum levels of IL-6 and CRP with consequent reduced concentrations of negative reactants of acute phase response (albumin, prealbumin, and transferrin) and increased concentrations of positive ones (haptoglobin and *α*_1_ globulin).

The patients also had increased blood concentrations of glucose and normal lactate concentrations. From a functional point of view, patients had severe disability (FIM −76.7% of normal value). At DOSS evaluation, nineteen patients had severe dysphagia (DOSS = 1.21 ± 0.88) and nineteen had a moderate dysphagia (DOSS = 3.07 ± 1.76). Daily calories and macronutrients administered or ingested were 22.4 ± 2.7 kcal/kg, 0.94 ± 0.17 g/kg protein, 2.5 ± 0.5 g/kg carbohydrates, and 0.98 ± 0.19 g/kg lipids. After randomization, the EAA and Plac subgroups were similar for all measured variables at baseline.

At discharge, both groups had similar BW reductions, which were not significantly different from the baseline, and similar improvements in the rate of dysphagia, physical disability, inflammation, and circulating proteins of acute phase response to inflammation. Blood glucose levels improved in the Plac group. Both groups had similar plasma lactate concentrations which over time did not differ from baseline values. The addition of EAAs 8 g/d EAAs to total protein administered/ingested (54.2 g/d) provided 6.9 g protein substrate [41]; so, at discharge, the treated group had had 1.02 kg protein provided.

## 4. Discussion

This study confirms the initial hypothesis that unaffected arm muscles of subacute stroke patients may have a prevalence of catabolic over anabolic activity. EAA supplements can potentially convert muscle hypercatabolism (MH) to anabolic/balanced protein metabolism. To the best of our knowledge, this is the first study to document both muscle hypercatabolism in dysphagic stroke patients at one month after acute event and the possibility of limiting/correcting it by supplemented essential amino acids.

### 4.1. Unaffected Arm MH at Patient Admission 

Persistent body inflammation, immobilization/disuse, and malnutrition were all factors present in the study population that can increase MH in the unaffected arm. The inflammatory status, primed by acute cerebrovascular accident [42] and possibly persisting over time by poststroke infarction complications [43], reduces protein synthesis and increases breakdown [44], also via IL-6 stimulated hypothalamus-pituitary cortico surrenal axis [45]. The rate of proteolysis was probably accentuated by insulin resistance as indicated in the study population and by blood glucose concentrations above the normal value. Inflammation was responsible for liver reprioritization of protein synthesis observed in the study patients.

Disuse, derived from immobilization, denervation, and muscle unloading, brings about increased proteolysis and, to a lesser extent, reduced protein synthesis [19]. Unloading* per se* may lead to muscle proteolysis via induced oxidative stress in skeletal muscle that triggers increased protein degradation [46]. Poststroke inadequate nutrition, in particular protein intake [47], contributes to proteolysis. Body weight loss and dysphagia reflect the fact that patients had had a prolonged inadequate nutritional intake before their admission to rehabilitation [18].

The MH finding seems to contrast with the normal levels of patients' circulating EAAs. This discrepancy can be reconciled by considering two factors; first, poor nutrition in acute setting would be due to inadequate energy intake but not protein intake, given that the former represents 89.6% of energy body requirements [48], whereas the latter is 99% of the recommended amount. The amounts of energy and protein ingested were similar to and, respectively, higher than those reported in a previous study conducted in stroke patients at similar period after acute event (21 d) [49]. Normal EAA levels suggest that 1 g/kg/d protein supply to/intake by subacute stroke in the rehabilitation stage of the disease may be nutritionally but not metabolically adequate to reduce muscle hypercatabolism. This would suggest that unaffected muscle is a site of profound metabolic perturbations, overriding EAA-promoted anabolic activity.

Disuse, unloading, and increased muscle cytokine content are some factors leading to MH.

Disuse activates the potent proteolytic activity of ATP-dependent ubiquitin-proteasome pathway, lysosomes, and calcium-dependent calpain system [50]. Unloading is a potent promoter of muscle proteolysis via inducing oxidative stress [46]. Increased muscle cytokine content may exert a proteolytic effect, in particular of myofibrillar protein [51].

Interestingly, cytokines affecting muscle cell function can be produced intrinsically within the muscle or by nonmuscle cells as neutrophils and macrophages [51]. During inflammation, these phagocytes infiltrate the muscular tissue [52]. Other nonresident cells such as fibroblasts [53, 54], vascular smooth muscle cells [55], and vascular endothelium [56] can produce cytokines.

Besides an adequate protein intake, reduced metabolic clearance of circulating BCAAs by the adipose tissue may contribute to normal arterial EAA levels. Indeed, adipose tissue modulates the levels of circulating BCAAs but in the case of insulin resistance, as in our study population, reduces or interrupts BCAA uptake [57].

Previous studies have addressed the timing of poststroke muscle loss in the unaffected limb.

Within the first week of stroke [58], one study found muscle weakness of unaffected quadriceps of hemiplegic stroke patients and a correlation between a change of quadriceps strength and acute weight loss [58]. Another investigation reported no evidence of muscle strength loss in any limb [59].

A number of studies have documented reduced muscle mass and strength six months after stroke [60]. This was more evident in the paretic compared with the nonparetic lower limb and upper limb. A study demonstrated reduced muscle strength in both legs in patients [61] one year after stroke compared to normal subjects [62].

This study adds information of the timing of the muscle loss of the unaffected limb as it documents muscle hypercatabolism in stroke patients one month after stroke. This would suggest that, in subacute stroke, systemic inflammatory-metabolic alterations may be an important contributor to muscle wasting, adding to other mechanisms of unaffected side weakness. These mechanisms include muscle damage from stroke lesion due to bilateral projections of each cerebral hemisphere [63], physical inactivity [14, 64], undernutrition, and possible motor weakness from comorbidities in preevent period. It is reasonable to believe that systemic factors also negatively impact the damaged controlateral muscles. Compared to healthy subjects, our patients also released significant amounts of the amino acids asparagine, threonine, and BCAAs. This would suggest progressive impoverishment of amino acid content of unaffected muscle.

Another finding differentiating stroke and healthy subjects is the concentration of certain amino acids in arterial blood. Strokes have decreased levels of aspartate, asparagine, glutamine, alanine, taurine, and tryptophan but increased levels of BCAAs, methionine, phenyl-, and lysine. In inflammation and muscle proteolysis, these reductions would suggest increased metabolic clearance of amino acids by visceral organs including liver, gut, and kidney, which would be in a hypermetabolic state. For instance, the liver has a high consumption of gluconeogenic aspartate, asparagine, alanine, and glutamine, the gut and the kidney of glutamine, the immune cells of glutamine, and the brain of all amino acids in particular of the serotonin precursor tryptophan.

The increases in arterial amino acid concentrations are mainly of muscular origin given that, in addition to phenyl-release, BCAAs and methionine, also lysine undergoes excessive release. The normal lactate levels suggest that, in unaffected arm muscles, there is a balanced aerobic-anaerobic pathway energy-forming. Interestingly, both protein degradation and synthesis require large amounts of energy in order to occur.

To sum up, this investigation shows that the unaffected arm muscles of hemiplegic, dysphagic stroke patients are sites of hypercatabolic activity which, if not corrected, leads to muscle wasting. Furthermore, dysphagic strokes have alterations of arterial amino acid profile. The loss of muscle mass and strength has a significant impact on stroke patients' functionality and life prognosis. Muscle depleted subjects have impaired glucose metabolic control [65, 66], increased risk of osteoporosis [64, 67], which may be responsible for hip-fracture and falls, cardiovascular deconditioning [64, 65, 67], and more accentuated disability, in particular walking [68]. Therefore, recognizing and treating muscle wasting as early as possible may be of paramount importance for rehabilitation outcome for stroke patients, especially if we consider that 80% of total neuromotor recovery occurs within the first month of acute stroke [69].

### 4.2. The Effects of EAA Supplementation on Unaffected Arm MH

This study clearly indicates that supplemented EAAs can correct unaffected muscle protein overdegradation in subacute dysphagic stroke patients. On the contrary, without EAA supplementation, patients continued to lose muscle mass seventy days after the acute stroke. After rehabilitation, the prevalently anabolic activity in the EAA group was accompanied by unaffected muscle uptake of 49% of arterial amino acids and of plasma total arterial amino acids suggesting the anabolic muscle protein turnover. This was reinforced by the lower releases of histidine, glycine, and taurine compared to those of the Plac group, as well as by increased arterial TAA availability. Anabolic activity associated with EAA supplementation has been documented both in experimental and in human studies. The main mechanisms involved are induced protein synthesis and reduced protein breakdown.

Regarding protein synthesis, essential BCAAs act as fuel and anabolic signals in human muscle [70]. Chronic supplementation of leucine, as mentioned here, stimulates postprandial protein synthesis in responsive tissues including skeletal muscle, liver, and adipose tissue [71]. Oral intake of 2.5 g leucine stimulates muscle protein synthesis after exercise or an overnight fast [72]. EAA ingestion stimulates muscle protein synthesis even in bedridden subjects [73] or during hypercortisolemia [73]. Amino acid infusion elevates plasma amino acids above base values leading to 30% increase in muscle protein synthesis of myofibrillar, sarcoplasmic, and mitochondrial proteins [74].

Regarding proteolysis, it is well documented that EAAs exert a potent antiproteolytic effect. The BCAA leucine is a regulating factor of myofibrillar protein degradation [75] as it suppresses myofibrillar protein degradation soon after oral administration. Infusion of BCAAs in humans markedly diminishes skeletal muscle protein degradation but stimulates protein synthesis in the heart [76, 77]. It has been shown that efficient protein use is determined by sensitivity variation of proteolysis to amino acids rather than protein synthesis [78]. Small amounts of amino acids are enough to reduce proteolysis unlike protein synthesis [78].

Several mechanisms underlie protein synthesis and, at the same time, reduce proteolysis by EAAs. One mechanism is the adequate availability of EAAs* per se*. Indeed, EAAs can stimulate protein synthesis independent of hormones [79]. Other mechanisms include the regulation of gene expression, modulation of anabolic hormone activities, improved aerobic metabolism energy-forming, and a reduced circulating TNF*α*/IGF-1 ratio. Finally, amino acids influence target genes at transcription, mRNA stability, and translation [80]. Amino acids and in particular EAAs promote protein synthesis by stimulating insulin-growth factor 1 (IGF-1) [81] and modulating insulin signalling [79]. Indeed, they play a role in regulating insulin signalling via the mTOR nutrient signalling pathway [79]. Insulin (and IGF-1) cannot stimulate protein synthesis if amino acid concentrations are not maintained [79].

Moreover, EAAs can also reduce insulin resistance [82]. EAAs induce anabolic activity indirectly by boosting cell aerobic metabolism to produce energy, the availability of which is indispensable for protein synthesis [24–27]. Particularly important in a state of systemic inflammation, EAAs can promote muscle protein synthesis by reducing circulating cytokine TNF*α*, so reducing the TNF*α*/IGF-1 index [31].

The discrepancy observed here between normal plasma EAA levels and muscle hypercatabolism suggests that chronic supplementation of free EAAs may be superior to protein EAAs in promoting muscle anabolism. Indeed, for muscle protein synthesis to occur, rapid increases of plasma EAA levels following EAA ingestion are more important than intramuscular amino acid availability [74]. This is because the protein synthetic machinery in muscle is unresponsive after 2.5 hrs [74]. The speed by which blood peak concentrations are achieved is higher after free EAA ingestion than after EAA from protein because the absorption rate of the latter is slowed by the copresence in the diet of complex carbohydrates and fats [83]. We showed that, in normal subjects, blood EAA concentrations after ingestion of 8 g free EAA were achieved within 40 minutes in young individuals and 90 minutes in elderly ones [84]. The modulation of muscle protein synthesis by blood elevations in EAAs may explain why, here, two stroke-patient groups had similar plasma EAA levels but different muscle protein metabolism responses.

A final reason why free EAAs are superior to EAA-protein in promoting anabolism is that free EAAs cause less efficient uptake of the splanchnic organs [85]. This has been indirectly confirmed here as we demonstrated that muscle anabolism following similar nutritional nitrogen availability occurred only in patients in which there was higher nitrogen availability from EAAs.

*Limitations of This Study*. Several study limitations require further investigations to overcome. One limit is the relatively small sample size of only dysphagic patients. A larger trial is also needed to document whether similar results are found in nondysphagic patients. We therefore consider this investigation as a pilot study. A comparative analysis between affected and unaffected arms would have strengthen the discussion and would have documented both the impact of systemic inflammation on metabolic alterations of denerved arm muscle and the rate of metabolic recovery, if any, exerted by supplemented EAAs.

The two subgroups of patients tended to reduce body weight during rehabilitation. This suggests a need to monitor energy intake/administered mainly in patients with increased energy expenditure after walking training protocols and/or recovery of walking capacity. Attention should also be paid to a possible synergistic effect between EAA supplementation and physical training of unaffected limbs. Patient body composition analysis would also have strengthened the discussion. Indeed, the evaluation of body tissue composition by dual X-ray absorptiometry (DEXA) or by bioelectrical impedance analysis (BIA) methods would have allowed us to measure the loss of fat-free mass (FFM) at rehabilitation admission and its time course during the hospital stay both for treated and placebo patients. In addition, measured FFM would have allowed us to provide patients with the same amount of EAAs per kg FFM.

We chose isoenergetic maltodextrin instead of nitrogen substance because it is well known that EAAs are superior to nitrogen from nonindispensable amino acids or from protein in promoting protein synthesis [86]. In addition, maltodextrin and EAAs share common immunological activity [87–89]. On the other hand, infection complications frequently follow acute strokes [90].

*Potential Clinical Implications*. Notwithstanding these limitations, this investigation provides some useful information for clinical practice. The physician should be aware of possible MH of the unaffected body side and of its reversibility. Indeed, recognizing and correcting MH as early as possible could allow patients better recovery in physical autonomy. This is a key point in reducing the risk of recurrent strokes, cardiovascular disease, metabolic syndrome, muscle atrophy, osteoporosis, and type II diabetes [91]. It is imperative that dysphagic stroke subjects are administered adequate amounts of high quality protein. It is useful to remember that we eat proteins but use their EAAs. Reduced protein intake leading to low blood EAA levels can contribute to a dramatic increase of MH. The amount of supplemented EAAs (8 g/d) is insufficient to enhance brain function recovery, given that the neurofunctional recovery was similar between both groups confirming the finding of a previous study [90].

## 5. Conclusions

This study shows the prevalence of muscle hypercatabolism of unaffected arm of dysphagic subacute stroke subjects. The hypercatabolism may be converted to anabolism by supplementing patients with free essential amino acid mixture.

## Figures and Tables

**Table 1:** Classification of stroke location, functional effects of stroke, and swallowing abnormalities of stroke population at admission to the rehabilitation centre.

	Patients (%)
Stroke location	
Cortical stroke:	
Dominant (left)	Number 9 (23.7%)
Nondominant (right)	Number 8 (21%)
Subcortical stroke:	
Dominant (left)	Number 7 (18.4%)
Nondominant (right)	Number 5 (13.2%)
Brainstem stroke	Number 6 (15.8%)
Cerebellar stroke	Number 3 (7.9%)

Stroke severity	
FIM impairments (score)	
Motor 21 ± 15^*^	
Cognitive 8 ± 6.5^*^	

Dysphagia (clinical/video fluoroscopic evaluation)	
Delayed oral transit	Number 18 (47.4%)
Incomplete oral clearance	Number 10 (26.3%)
Wet voice	Number 3 (7.9%)
Absent cough	Number 7 (18.4%)

FIM: functional independence measure. ^*^Total FIM scores range from 18 (totally dependent) to 126 (totally independent); motor scores range from 13 (total dependence) to 91 (total independence); cognitive scores range from 5 (total dependence) to 35 (total independence).

**Table 2:** Nutrition composition of an individual packet of supplements, containing 4 g of an amino acid mixture, used in this study^a^.

kcal	21.9
kJ	91.7
Proteins	0 g
Carbohydrates	1.046 g
Fats	0.074 g
Total amino acids, including the following:	4 g
L-leucine	1250 mg
L-lysine	650 mg
L-isoleucine	625 mg
L-valine	625 mg
L-threonine	350 mg
L-cysteine	150 mg
L-histidine	150 mg
L-phenylalanine	100 mg
L-methionine	50 mg
L-tyrosine	30 mg
L-tryptophan	20 mg
Vitamin B6	0.15 mg
Vitamin B1	0.15 mg

^a^Treated patients were given 2 packets daily (8 g essential amino acids).

**Table 3:** Amino acid arterial concentrations and A − V differences found in stroke patients and matched healthy subjects.

Amino acid profiles (*µ*mol/L)	Healthy subjects (*n* = 8)	Stroke (*n* = 38)	*P* value
Aspartate			
A	98.1 ± 40.6	16.6 ± 6.7	*P* = 0.001
A − V	− 0.3 ± 14.3	0.55 ± 4.7	*P* = 0.9
Glutamate			
A	198.7 ± 10.6	195.5 ± 137.3	*P* = 0.9
A − V	− 7.5 ± 21	− 6.5 ± 55.9	*P* = 0.8
Histidine			
A	58 ± 5.1	55.5 ± 10.3	*P* = 0.7
A − V	− 0.4 ± 5	− 6.1 ± 8.7	*P* = 0.3
Asparagine			
A	61 ± 1.1	35.5 ± 10.5	*P* < 0.001
A − V	4.9 ± 5.5	− 4.3 ± 6.6	*P* = 0.002
Serine			
A	88.4 ± 4.3	108 ± 35.7	*P* = 0.025
A − V	− 2.4 ± 6.4	1.5 ± 24.6	*P* = 0.6
Glutamine			
A	464.8 ± 14	323.5 ± 184.2	*P* = 0.003
A − V	− 2.4 ± 23	− 18.2 ± 67.2	*P* = 0.2
Arginine			
A	59.3 ± 7.6	89.1 ± 69.42	*P* = 0.6
A − V	7.3 ± 19.5	18 ± 62.4	*P* = 0.3
Citrulline			
A	24.2 ± 3.8	30.3 ± 14	*P* = 0.7
A − V	− 0.9 ± 5.6	1.2 ± 7.3	*P* = 0.5
Glycine			
A	268.3 ± 12	239.3 ± 60.9	*P* = 0.8
A − V	9.9 ± 29.9	− 19.4 ± 41.8	*P* = 0.2
^*^Threonine			
A	106.6 ± 11	120.4 ± 41.5	*P* = 0.8
A − V	− 0.8 ± 14.8	− 12.2 ± 10.5	*P* = 0.029
Alanine			
A	312.6 ± 15.7	259.3 ± 84	*P* = 0.012
A − V	− 15 ± 20.5	− 68.5 ± 40.4	*P* = 0.002
Taurine			
A	125.8 ± 9.7	55.6 ± 23.1	*P* < 0.001
A − V	8.1 ± 12.9	− 34 ± 23	*P* < 0.001
Tyrosine			
A	56.3 ± 6.1	57.4 ± 21.3	*P* = 0.9
A − V	4.5 ± 9.1	− 2.75 ± 11	*P* = 0.4
^∗°^Valine			
A	155 ± 12.5	229.4 ± 47.7	*P* = 0.005
A − V	4.4 ± 18.9	− 15.5 ± 16.6	*P* = 0.064
^*^Methionine			
A	10.75 ± 1.7	35.1 ± 6.6	*P* < 0.001
A − V	0.7 ± 1.9	− 0.3 ± 4.9	*P* = 0.5
^*^Tryptophan			
A	51.1 ± 4.6	33.9 ± 7.8	*P* < 0.001
A − V	− 0.5 ± 7.6	− 3.7 ± 4.1	*P* = 0.5
^*^Phenylalanine			
A	46.3 ± 5.7	67.4 ± 26.6	*P* = 0.037
A − V	0.3 ± 6.6	− 6.9 ± 8.1	*P* < 0.03
^∗°^Isoleucine			
A	45.8 ± 5	76 ± 17.7	*P* < 0.001
A − V	1.1 ± 5.7	− 6.4 ± 8.5	*P* = 0.5
^∗°^Leucine			
A	78.13 ± 6.35	135.6 ± 36.4	*P* < 0.001
A − V	0.38 ± 7.2	− 18.8 ± 33.3	*P* = 0.02
Ornithine			
A	56.4 ± 6.4	60.9 ± 20.9	*P* = 0.8
A − V	1 ± 10.5	− 10.1 ± 8.7	*P* = 0.01
^*^Lysine			
A	115.8 ± 11	201.4 ± 71.6	*P* < 0.001
A − V	− 1.3 ± 16.7	− 12.6 ± 53	*P* = 0.4
Total amino acids			
A	24.81 ± 60.5	2425.7 ± 601	*P* = 0.2
A − V	13.24 ± 78.1	− 225 ± 267.6	*P* = 0.6
^*^EAAs			
A	609.4 ± 18.9	899.2 ± 194.1	*P* = 0.5
A − V	4.3 ± 21.2	− 76.4 ± 167	*P* = 0.3
°BCAAs			
A	279 ± 13.2	441 ± 88.4	*P* = 0.2
A − V	5.9 ± 25.7	− 40.7 ± 59	*P* = 0.09

Data are expressed as mean ± standard deviation (SD). Statistical analysis: unpaired *t*-test.

^*^Essential amino acids (EAAs); °branched chain amino acids (BCAAs).

**Table 4:** Amino acid arterial concentrations and A − V differences found in the two patient subgroups randomized to receive treatment (EAAs) or placebo, both at admission and discharge from rehabilitation.

Amino acid profiles (*µ*mol/L)	Admission			Discharge		^∧^Trend over time (*P* level) interaction
	Placebo (*n* = 19)	EAAs (*n* = 19)	^§^ *P* value	Placebo (*n* = 19)	EAAs (*n* = 19)	
Aspartate						
A	16.27 ± 7.3	17.11 ± 6.4	*P* = 0.1	17.3 ± 6	17.5 ± 10.6	*P* = 0.1
A − V	1 ± 5.2	0.01 ± 4.3	*P* = 0.2	− 2 ± 4.3	2.8 ± 5.5	*P* = 0.04
Glutamate						
A	207 ± 153.5	181.6 ± 122	*P* = 0.2	171.1 ± 102.3	150 ± 102.8	*P* = 0.3
A − V	− 12.7 ± 71.3	1.1 ± 31	*P* = 0.5	− 14.7 ± 35	4.7 ± 37.4	*P* = 0.8
Histidine						
A	59.36 ± 10.8	50.9 ± 8	*P* = 0.1	59.2 ± 11.8	68.9 ± 13.6	*P* = 0.7
A − V	− 4.6 ± 9.3	− 8 ± 8	*P* = 0.4	− 7.7 ± 4.8	− 1.4 ± 6.2	*P* = 0.02
Asparagine						
A	39 ± 7.7	38 ± 13.5	*P* = 0.1	35.4 ± 5.4	43.1 ± 10.4	*P* = 0.05
A − V	− 2.8 ± 5.4	− 6 ± 7.8	*P* = 0.7	− 5.2 ± 3.5	− 1.1 ± 4.2	*P* = 0.03
Serine						
A	115 ± 42.9	99.3 ± 24.1	*P* = 0.1	107.8 ± 30.2	119 ± 40.6	*P* = 0.9
A − V	0.9 ± 25	2.22 ± 25.5	*P* = 0.6	− 1.3 ± 19.6	15.2 ± 31.1	*P* = 0.5
Glutamine						
A	323.3 ± 192.8	323.8 ± 184.8	*P* = 0.4	378 ± 131.6	463.6 ± 99	*P* = 0.4
A − V	6.6 ± 75.5	− 48.4 ± 41.7	*P* = 0.07	− 10.8 ± 69.6	− 22.6 ± 31.6	*P* = 0.07
3-Methylhistidine						
A	3 ± 1.2	2.2 ± 1	*P* = 0.2	2.4 ± 1.4	2.4 ± 1.1	*P* = 0.2
A − V	0.3 ± 1.1	− 0.1 ± 0.1	*P* = 0.2	− 0.2 ± 1.1	− 0.03 ± 1.1	*P* = 0.1
Arginine						
A	103.7 ± 86.2	71.2 ± 38.8	*P* = 0.3	98.4 ± 57.1	104.9 ± 65.2	*P* = 0.08
A − V	30.6 ± 81.6	2.7 ± 20.9	*P* = 0.5	10.8 ± 61.7	40.4 ± 62.1	*P* = 0.07
Citrulline						
A	33.7 ± 12.2	26.1 ± 15.7	*P* = 0.2	35.7 ± 13.7	35.6 ± 21.1	*P* = 0.4
A − V	2.3 ± 9.6	− 0.2 ± 3.2	*P* = 0.5	− 3 ± 5.6	0.3 ± 1.3	*P* = 0.09
Glycine						
A	243.6 ± 71.7	233.9 ± 48.1	*P* = 0.2	240.9 ± 40.4	308.1 ± 9	*P* = 0.08
A − V	− 7.4 ± 48.9	− 34 ± 26.8	*P* = 0.8	− 38.1 ± 21.3	− 9.3 ± 24.5	*P* = 0.01
^*^Threonine						
A	113.3 ± 19.5	129 ± 58.9	*P* = 0.2	131.8 ± 52.6	157.7 ± 54.6	*P* = 0.5
A − V	− 11.5 ± 8.6	− 13 ± 12.9	*P* = 0.2	− 10.5 ± 16.2	2.4 ± 15.5	*P* = 0.09
Alanine						
A	242.3 ± 78.2	280.1 ± 90.8	*P* = 0.1	274.7 ± 76.1	365.5 ± 61.6	*P* = 0.6
A − V	− 70.5 ± 27	− 66 ± 54.4	*P* = 0.2	− 58.9 ± 68.7	− 33 ± 50.2	*P* = 0.08
Taurine						
A	61.8 ± 20.7	4 ± 24.8	*P* = 0.08	43.7 ± 5.9	37.3 ± 16.2	*P* = 0.09
A − V	− 33 ± 29.5	− 35.3 ± 13.2	*P* = 0.3	− 52 ± 21.4	− 27.1 ± 13.6	*P* = 0.006
Tyrosine						
A	56.5 ± 10.9	58.4 ± 30.4	*P* = 0.1	49.2 ± 14.8	62.1 ± 27.2	*P* = 0.8
A − V	− 4.5 ± 5.5	− 0.7 ± 15.6	*P* = 0.4	− 4.8 ± 2.7	1.9 ± 7.8	*P* = 0.03
^∗°^Valine						
A	265.7 ± 39.8	202.3 ± 33.7	*P* = 0.4	198.7 ± 49.1	248.5 ± 31.8	*P* = 0.6
A − V	− 12.5 ± 20.5	− 17 ± 17.6	*P* = 0.07	− 18 ± 12.7	0.05 ± 21.2	*P* = 0.7
^*^Methionine						
A	36 ± 8.7	34.5 ± 5.9	*P* = 0.2	27.7 ± 16.8	26 ± 2.8	*P* = 0.5
A − V	0.5 ± 0.7	− 0.8 ± 6.3	*P* = 0.3	3.5 ± 14.8	− 2 ± 2.8	*P* = 0.7
^*^Tryptophan						
A	35 ± 8.1	32.4 ± 7.7	*P* = 0.1	33.1 ± 8.4	36.1 ± 9.1	*P* = 0.8
A − V	− 3.1 ± 4.6	− 4.3 ± 3.7	*P* = 0.2	− 3.3 ± 3.3	− 0.4 ± 6.1	*P* = 0.3
^*^Phenylalanine						
A	69.7 ± 21.9	64.4 ± 32.5	*P* = 0.1	51.4 ± 11.1	56.7 ± 11.8	*P* = 0.5
A − V	− 6.9 ± 7.6	− 6.8 ± 9.1	*P* = 0.1	− 6 ± 4.4	0.9 ± 7.6	*P* = 0.02
^∗°^Isoleucine						
A	81.9 ± 12.2	68.8 ± 21.2	*P* = 0.3	85.4 ± 65.6	111.5 ± 73.1	*P* = 0.09
A − V	− 6.4 ± 22	− 6.3 ± 14.5	*P* = 0.1	6 ± 28.3	11.6 ± 17.6	*P* = 0.5
^∗°^Leucine						
A	148.6 ± 30.8	119.6 ± 38	*P* = 0.07	149.6 ± 134.2	191.7 ± 119.1	*P* = 0.4
A − V	− 13.8 ± 28	− 24.2 ± 39.2	*P* = 0.5	7.7 ± 16.4	11.7 ± 26.4	*P* = 0.8
Ornithine						
A	63.7 ± 12.6	58.1 ± 27.4	*P* = 0.5	56 ± 13.9	57.3 ± 22.8	*P* = 0.8
A − V	− 13.4 ± 9.3	− 6.7 ± 7	*P* = 0.09	− 17.6 ± 17.6	− 2.1 ± 21	*P* = 0.08
^*^Lysine						
A	205.3 ± 77	196.7 ± 68.6	*P* = 0.7	209.6 ± 95.6	248.2 ± 124	*P* = 0.8
A − V	− 8.3 ± 67.2	− 17.8 ± 31.3	*P* = 0.6	− 1.9 ± 64.3	58.3 ± 122.1	*P* = 0.7
Total-amino acids						
A	2523.7 ± 331.8	2292.4 ± 472.7	*P* = 0.3	2457.1 ± 826.8	2747 ± 465.9	*P* = 0.02
A − V	− 169.2 ± 25.6	− 289.6 ± 18	*P* = 0.5	− 63.8 ± 25.7	51.2 ± 23.5	*P* = 0.05
^*^EAAs						
A	956 ± 103	848 ± 242	*P* = 0.5	887.3 ± 254	1076.4 ± 295	*P* = 0.05
A − V	− 62 ± 20	− 90.2 ± 16.9	*P* = 0.3	− 22.5 ± 20	82.6 ± 27.4	*P* = 0.01
°BCAAs						
A	493.6 ± 57.3	391 ± 84.5	*P* = 0.5	433.7 ± 213.9	552 ± 225.9	*P* = 0.09
A − V	− 32.7 ± 23.5	− 47.5 ± 23.8	*P* = 0.6	− 4.3 ± 19	23.35 ± 21.7	*P* = 0.05

Data are expressed as mean ± standard deviation (SD). Statistical analysis: ^§^unpaired *t*-test; ^∧^repeated measures analysis of variance. Trend over time: interaction differences in trends between groups. ^*^Essential amino acids (EAAs); °branched chain amino acids (BCAAs).

**Table 5:** Demographic, anthropometric, biohumoral, and neurofunctional characteristics and nutritional intake/supply of stroke patients as an entire group, EAA and placebo subgroups, both at admission and discharge.

Variables	nv	All patients (*n* = 38)		Placebo (*n* = 19)		EAAs (*n* = 19)		Trend over time (*P* level) interaction
		Admission	Discharge	Admission	Discharge	Admission	Discharge	
Demographic								
Male/female	—	25/13	—	13/6	—	12/7	—	—
Age (years)	—	69.7 ± 11.4	—	71.3 ± 10	—	68 ± 13.2	—	—
Anthropometric								
Actual body weight (kg)	—	59.8 ± 10.2	57.7 ± 9.8	57.6 ± 7.1	55.9 ± 7.5	62.2 ± 12.7	59.7 ± 12	*P* = 0.6
Actual/habitual body weight (%)	—	90.4 ± 7.4	87.5 ± 9.5	90.5 ± 7.2	87.8 ± 9.3	90.3 ± 8	87.1 ± 10.3	*P* = 0.8
BMI (kg/m^2^)	—	21.6 ± 3	20.88 ± 3.14	21.3 ± 2.6	20.7 ± 2.9	22 ± 3.5	21.1 ± 3.5	*P* = 0.8
Blood								
ESR 1st hr (mm)	2–20	35.8 ± 11.7	33.2 ± 18.1	31.2 ± 6.8	27.6 ± 16	43.3 ± 15.8	37.2 ± 19.7	*P* = 0.7
Haemoglobin (g/dL)	F > 12; M > 13	12.2 ± 1.6	12.1 ± 1.2	12.6 ± 1.8	12.3 ± 1.3	11.7 ± 1	12 ± 1.1	*P* = 0.07
Blood urea (mg/dL)	20–40	42.7 ± 19.7	37.5 ± 19	48.70 ± 15	37.6 ± 7.6	32.7 ± 23.7	37.3 ± 26	*P* = 0.9
Serum creatinine (mg/dL)	0.7–1.2	1 ± 0.3	1 ± 0.3	1.1 ± 0.4	1.1 ± 0.3	0.8 ± 0.2	0.9 ± 0.3	*P* = 0.5
Plasma glucose (mg/dL)	80–110	115.1 ± 23.6	106.9 ± 11.6	122.5 ± 25.8	104.8 ± 14.1	100.3 ± 8.5	109.7 ± 9.3	*P* = 0.07
Interleukin-6 (pg/mL)	<7	15.9 ± 14.9	6.7 ± 9.86	11.7 ± 9	8.5 ± 13.5	19.6 ± 18.5	4.6 ± 2.3	*P* = 0.5
Serum C-reactive protein (CRP) (mg/dL)	<0.3	1.9 ± 1.9	1.4 ± 2.5	1.7 ± 1.3	1.6 ± 2.8	2.2 ± 2.5	1.2 ± 2.3	*P* = 0.6
Fibrinogen (mg/dL)	230–550	452.4 ± 75.8	400.9 ± 87.9	465.1 ± 87.3	387.2 ± 68.2	438 ± 63.1	412.7 ± 106	*P* = 0.8
Serum haptoglobin (mg/dL)	30–200	293.6 ± 93	211 ± 75	313 ± 94	245 ± 74	272 ± 92	169 ± 56	*P* = 0.7
Serum *α*1 globulin (mg/dL)	210–350	504 ± 74	436 ± 89	506 ± 80	467 ± 92	472 ± 79	382 ± 85	*P* = 0.8
Serum albumin (g/dL)	4.02–4.76	2.9 ± 0.5	3.2 ± 0.5	3 ± 0.5	3.1 ± 0.5	2.7 ± 0.6	3.3 ± 0.5	*P* = 0.03
Serum prealbumin (mg/dL)	18–30	18.8 ± 5.7	20.9 ± 7.1	19.4 ± 6.5	19 ± 5.9	18.1 ± 4.9	22.9 ± 8.1	*P* = 0.7
Serum transferrin (mg/dL)	202–364	183.1 ± 28.3	193.8 ± 35.1	186.1 ± 33.2	195.4 ± 39.6	179.3 ± 22.3	192 ± 31.8	*P* = 0.5
Plasma lactate (mmol/L)	0.6–2.2	1.6 ± 0.5	2 ± 0.6	1.4 ± 0.4	2 ± 0.6	1.8 ± 0.5	2 ± 0.5	*P* = 0.3
Neurofunctional								
FIM score	125	29.4 ± 18.5	54 ± 31.2	31.1 ± 16	60 ± 36.8	27.6 ± 21.7	47.4 ± 23.9	*P* = 0.5
DOSS score	1–7	2.1 ± 1.3	3.3 ± 1.7	2.5 ± 1.3	3.9 ± 1.8	1.6 ± 1.3	2.6 ± 1.5	*P* = 0.7
Nutrition (PEG or oral intake)								
Energy								—
(kcal/d)	—	1293.6 ± 155	Same	1362 ± 143	Same	1293 ± 155	Same	
(kcal/kg)	≥25	22.4 ± 2.7		23.6 ± 2.5		20.7 ± 2.9		
Protein								—
(g/d)	—	54.1 ± 9.6	Same	58.7 ± 10.2	Same	54.2 ± 9.6	Same	
(g/kg)	≥1.1	0.94 ± 0.17		1.02 ± 0.17		0.87 ± 0.19	1.02 ± 0.20^*^	
Carbohydrates								—
(g/d)	—	146.2 ± 32	Same	164.3 ± 30.1	Same	146.2 ± 32	Same	
(g/kg)	2.5–4	2.5 ± 0.5		2.85 ± 0.2		2.35 ± 0.9		
Lipids								—
(g/d)	—	56.9 ± 11	Same	55.3 ± 5.5	Same	50.1 ± 7.5	Same	
(g/kg)	≤1	0.98 ± 0.19		1 ± 0.1		0.8 ± 0.1		

Data are expressed as mean ± standard deviation (SD). Statistical analysis: repeated measures analysis of variance. Trend over time: interaction differences in trends between groups.

BMI: body mass index; ESR: erythrocyte sedimentation rate; FIM: functional independence measure; DOSS: Dysphagia Outcome and Severity Scale.

^*^This amount is the sum of the protein administered/ingested (54.2 g) and protein (6.9 g) provided by supplemented EAAs [41].
